# Supplementary material for: A Likelihood Approach for Real-Time Calibration of Stochastic Compartmental Epidemic Models
Source: PLoS Comput Biol. 2017 Jan 17;13(1):e1005257. doi: 10.1371/journal.pcbi.1005257 (PMC5240920; doi:10.1371/journal.pcbi.1005257)
Supplement: S1 File — (TAR.GZ) [file pcbi.1005257.s014.tar.gz › HSPH_Online-SI-Revision/IPoi09/n10-extreme/n10-extreme_BENCH2_table-8.pdf]

1	newly 3 weeks spec	116.	396.375	{25., 937.}	271.967	0.661643	newly 3 weeks cumul	174.	677.804	{38., 1636.}	317.457	0.695097	newly 5 weeks spec	547.	902.679	{112., 1560.}	96.6701	0.496661	newly 5 weeks cumul	988.	2254.73	{194., 4376.}	158.312	0.602329	newly 66 weeks cumul	9069.	7711.68	{4744., 9288.}	15.9472	0.320895
2	newly 3 weeks spec	211.	414.958	{91., 627.}	117.239	0.481972	newly 3 weeks cumul	450.	1142.08	{153., 1809.}	174.741	0.589478	newly 5 weeks spec	408.	324.042	{113., 472.}	32.4857	0.328233	newly 5 weeks cumul	1168.	1853.25	{681., 2607.}	77.9324	0.428215	newly 62 weeks cumul	7262.	3024.92	{1984., 4148.}	58.346	0.560829
3	newly 3 weeks spec	63.	13.44	{0., 24.}	95.873	1.91296	newly 3 weeks cumul	84.	21.26	{0., 40.}	93.5952	1.65701	newly 5 weeks spec	540.	86.86	{0., 172.}	95.7074	2.52141	newly 5 weeks cumul	830.	148.02	{0., 260.}	95.6627	2.30319	newly 68 weeks cumul	9108.	3815.73	{0., 8989.}	58.4213	2.37911
4	newly 3 weeks spec	319.	435.352	{65., 884.}	80.5933	0.35047	newly 3 weeks cumul	641.	918.778	{131., 1947.}	90.9227	0.377473	newly 5 weeks spec	626.	688.093	{198., 996.}	41.7732	0.218941	newly 5 weeks cumul	1754.	2173.11	{476., 3784.}	62.3971	0.291452	newly 62 weeks cumul	8332.	7918.33	{6185., 9219.}	12.2642	0.0597835
5	newly 3 weeks spec	240.	647.681	{5., 1201.}	194.707	0.704967	newly 3 weeks cumul	440.	1467.4	{14., 3473.}	258.946	0.779436	newly 5 weeks spec	689.	800.255	{0., 1355.}	54.2167	0.57027	newly 5 weeks cumul	1573.	3035.13	{14., 5240.}	117.888	0.678454	newly 65 weeks cumul	9428.	5930.49	{14., 9149.}	37.0971	0.642961
6	newly 3 weeks spec	128.	584.547	{92., 1162.}	373.511	0.743159	newly 3 weeks cumul	239.	928.283	{138., 1936.}	308.108	0.700064	newly 5 weeks spec	489.	1368.04	{659., 1869.}	191.083	0.632486	newly 5 weeks cumul	1036.	3338.34	{1184., 5319.}	234.705	0.689786	newly 66 weeks cumul	9287.	7933.11	{6173., 9277.}	14.7242	0.326337
7	newly 3 weeks spec	131.	97.4407	{0., 320.}	99.4954	1.27262	newly 3 weeks cumul	207.	169.746	{0., 459.}	99.2385	1.19095	newly 5 weeks spec	469.	287.983	{0., 807.}	83.8784	1.48939	newly 5 weeks cumul	924.	641.78	{0., 2057.}	90.1185	1.38857	newly 67 weeks cumul	8335.	5420.19	{0., 9317.}	40.3978	1.34447
8	newly 3 weeks spec	187.	644.282	{197., 1115.}	256.27	0.5968	newly 3 weeks cumul	315.	1338.85	{317., 2423.}	336.423	0.662301	newly 5 weeks spec	589.	982.155	{678., 1269.}	77.7876	0.374976	newly 5 weeks cumul	1255.	3166.35	{1443., 4922.}	163.542	0.522275	newly 64 weeks cumul	9223.	8172.42	{7228., 9089.}	11.4299	0.180927
9	newly 3 weeks spec	245.	466.538	{108., 924.}	118.003	0.389568	newly 3 weeks cumul	451.	900.523	{182., 1902.}	129.282	0.401665	newly 5 weeks spec	556.	884.969	{338., 1223.}	76.4278	0.316209	newly 5 weeks cumul	1389.	2472.43	{796., 4266.}	98.724	0.36273	newly 64 weeks cumul	8874.	8507.2	{7762., 9220.}	6.58068	0.0990443
10	newly 3 weeks spec	219.	784.196	{432., 1213.}	268.341	0.611274	newly 3 weeks cumul	414.	1841.	{787., 2911.}	355.352	0.69901	newly 5 weeks spec	476.	847.294	{281., 1245.}	99.3492	0.379506	newly 5 weeks cumul	1223.	3564.49	{2164., 5132.}	201.501	0.549871	newly 64 weeks cumul	8927.	6394.25	{3587., 8659.}	28.4499	0.254714
11	newly 3 weeks spec	260.	182.531	{31., 342.}	45.9655	0.369292	newly 3 weeks cumul	477.	418.49	{69., 825.}	54.6229	0.363428	newly 5 weeks spec	539.	305.469	{51., 517.}	44.1899	0.377851	newly 5 weeks cumul	1360.	965.531	{171., 1722.}	43.8851	0.349301	newly 59 weeks cumul	8770.	7137.08	{5715., 8118.}	18.6194	0.0941685
12	newly 3 weeks spec	147.	149.762	{14., 307.}	74.2468	0.489447	newly 3 weeks cumul	271.	280.968	{22., 596.}	75.675	0.470172	newly 5 weeks spec	376.	375.667	{58., 734.}	60.1275	0.423777	newly 5 weeks cumul	836.	900.333	{110., 1813.}	69.4672	0.430335	newly 64 weeks cumul	8374.	8011.02	{5389., 9249.}	11.5676	0.112088
13	newly 3 weeks spec	356.	213.304	{49., 471.}	52.3325	0.436848	newly 3 weeks cumul	666.	447.357	{97., 1016.}	54.0165	0.432288	newly 5 weeks spec	681.	398.571	{123., 709.}	43.9165	0.373926	newly 5 weeks cumul	1797.	1143.79	{304., 2377.}	47.9331	0.401623	newly 60 weeks cumul	8214.	7925.52	{6823., 8782.}	8.45964	0.1178
14	newly 3 weeks spec	267.	741.552	{200., 1187.}	192.238	0.459016	newly 3 weeks cumul	466.	1400.84	{307., 2373.}	217.06	0.478494	newly 5 weeks spec	683.	1201.12	{682., 1530.}	83.9224	0.276554	newly 5 weeks cumul	1583.	3638.79	{1642., 5194.}	139.663	0.38337	newly 65 weeks cumul	8671.	8470.55	{7002., 9220.}	7.69469	0.0364209
15	newly 3 weeks spec	284.	325.278	{57., 607.}	69.6792	0.345169	newly 3 weeks cumul	552.	732.861	{118., 1438.}	83.2579	0.354752	newly 5 weeks spec	600.	484.583	{106., 893.}	42.875	0.289749	newly 5 weeks cumul	1572.	1620.72	{324., 2906.}	55.1527	0.301318	newly 62 weeks cumul	7388.	5950.94	{3486., 8747.}	27.022	0.156097
16	newly 3 weeks spec	140.	102.985	{10., 273.}	68.6141	0.592692	newly 3 weeks cumul	241.	181.104	{20., 444.}	69.5176	0.540936	newly 5 weeks spec	517.	308.328	{28., 719.}	60.0508	0.570036	newly 5 weeks cumul	1029.	675.493	{71., 1665.}	62.7286	0.559456	newly 65 weeks cumul	8875.	8336.45	{7426., 9300.}	9.29712	0.16359
17	newly 3 weeks spec	117.	62.5085	{0., 175.}	87.5996	1.22533	newly 3 weeks cumul	203.	101.542	{0., 300.}	84.7625	1.18314	newly 5 weeks spec	499.	238.695	{0., 667.}	78.7813	1.34873	newly 5 weeks cumul	983.	473.475	{0., 1461.}	80.8214	1.33305	newly 67 weeks cumul	8786.	5986.93	{0., 9176.}	34.0123	1.14337
18	newly 3 weeks spec	167.	471.381	{105., 950.}	197.966	0.440935	newly 3 weeks cumul	309.	823.143	{147., 1679.}	183.916	0.416405	newly 5 weeks spec	657.	1074.21	{519., 1407.}	75.6058	0.263857	newly 5 weeks cumul	1340.	2679.43	{933., 4418.}	113.93	0.334476	newly 65 weeks cumul	9346.	8227.44	{6321., 9266.}	11.9822	0.0616115
19	newly 3 weeks spec	61.	44.9265	{0., 133.}	102.001	1.17331	newly 3 weeks cumul	87.	71.1618	{0., 198.}	101.471	0.97819	newly 5 weeks spec	390.	194.75	{0., 646.}	87.2587	1.49698	newly 5 weeks cumul	643.	365.294	{0., 1090.}	90.687	1.29916	newly 67 weeks cumul	8626.	6357.75	{0., 9259.}	31.4458	1.11141
20	newly 3 weeks spec	181.	135.492	{23., 299.}	57.3396	0.428428	newly 3 weeks cumul	336.	250.785	{42., 517.}	56.4148	0.428648	newly 5 weeks spec	555.	365.862	{75., 645.}	46.0762	0.376261	newly 5 weeks cumul	1224.	846.508	{159., 1572.}	49.8052	0.402154	newly 64 weeks cumul	9145.	8153.34	{7123., 9326.}	11.5517	0.127553
21	newly 3 weeks spec	233.	322.153	{83., 601.}	76.8604	0.336966	newly 3 weeks cumul	468.	701.051	{179., 1277.}	88.1863	0.358135	newly 5 weeks spec	546.	552.068	{221., 852.}	37.5861	0.241384	newly 5 weeks cumul	1440.	1690.39	{516., 2846.}	56.529	0.305596	newly 61 weeks cumul	8562.	8027.32	{7144., 8930.}	8.34115	0.114324
22	newly 3 weeks spec	42.	10.74	{0., 24.}	98.5714	1.79387	newly 3 weeks cumul	65.	17.16	{0., 36.}	95.1385	1.61422	newly 5 weeks spec	339.	59.13	{0., 120.}	93.9263	2.43569	newly 5 weeks cumul	532.	101.57	{0., 204.}	94.3102	2.18266	newly 68 weeks cumul	9331.	3412.51	{0., 9026.}	63.4553	2.4566
23	newly 3 weeks spec	289.	469.385	{145., 737.}	86.3455	0.4057	newly 3 weeks cumul	567.	1267.35	{263., 2094.}	144.356	0.520277	newly 5 weeks spec	502.	418.077	{80., 779.}	49.3411	0.40857	newly 5 weeks cumul	1436.	2157.5	{723., 3096.}	72.0511	0.399414	newly 63 weeks cumul	6906.	3435.96	{1893., 5093.}	50.2467	0.484598
24	newly 3 weeks spec	396.	561.371	{144., 944.}	72.1934	0.28346	newly 3 weeks cumul	794.	1210.63	{271., 2312.}	85.4048	0.316225	newly 5 weeks spec	721.	850.043	{428., 1061.}	34.9851	0.159375	newly 5 weeks cumul	2068.	2792.14	{965., 4265.}	59.3755	0.239225	newly 61 weeks cumul	8926.	8812.19	{8304., 9263.}	3.33456	0.0148108
25	newly 3 weeks spec	77.	7.5098	{0., 19.}	90.247	1.45821	newly 3 weeks cumul	116.	17.6863	{2., 49.}	84.7532	1.12639	newly 5 weeks spec	441.	13.3333	{0., 33.}	96.9766	2.01619	newly 5 weeks cumul	781.	40.1176	{2., 104.}	94.8633	1.64927	newly 64 weeks cumul	9000.	5383.69	{2., 8183.}	40.1813	0.879422
26	newly 3 weeks spec	130.	461.552	{0., 1186.}	301.653	0.854688	newly 3 weeks cumul	214.	662.119	{0., 1692.}	258.39	0.836386	newly 5 weeks spec	577.	1270.43	{0., 2091.}	152.497	0.793322	newly 5 weeks cumul	1076.	2864.48	{0., 6030.}	201.9	0.859143	newly 67 weeks cumul	7335.	7553.42	{0., 9373.}	30.6805	0.637547
27	newly 3 weeks spec	133.	115.8	{3., 295.}	77.995	0.657418	newly 3 weeks cumul	225.	205.4	{7., 566.}	80.5481	0.619511	newly 5 weeks spec	482.	332.583	{17., 719.}	60.944	0.620638	newly 5 weeks cumul	995.	744.583	{43., 1737.}	67.8174	0.640751	newly 65 weeks cumul	9083.	8096.73	{6807., 9292.}	12.0004	0.266404
28	newly 3 weeks spec	69.	539.53	{0., 2679.}	800.391	1.92038	newly 3 weeks cumul	105.	964.46	{0., 4784.}	936.305	1.94775	newly 5 weeks spec	530.	478.67	{0., 1759.}	115.666	2.12534	newly 5 weeks cumul	861.	1987.73	{0., 7737.}	253.107	2.28329	newly 68 weeks cumul	9312.	3939.03	{0., 9095.}	57.7069	2.48458
29	newly 3 weeks spec	156.	36.0656	{0., 80.}	76.9441	1.06107	newly 3 weeks cumul	228.	76.8852	{3., 166.}	68.608	0.866234	newly 5 weeks spec	612.	84.8361	{0., 190.}	86.1379	1.29957	newly 5 weeks cumul	1151.	218.902	{4., 467.}	80.9816	1.1441	newly 63 weeks cumul	9014.	7200.13	{6043., 8732.}	20.1228	0.503679
30	newly 3 weeks spec	209.	428.582	{111., 894.}	127.873	0.354258	newly 3 weeks cumul	375.	846.672	{207., 1862.}	148.681	0.37603	newly 5 weeks spec	521.	782.075	{357., 1100.}	65.3508	0.236619	newly 5 weeks cumul	1271.	2241.79	{767., 3962.}	95.7161	0.299389	newly 63 weeks cumul	8143.	8793.31	{7965., 9300.}	10.2476	0.0432601
31	newly 3 weeks spec	173.	704.71	{67., 1423.}	328.843	0.726034	newly 3 weeks cumul	303.	1130.81	{105., 2332.}	297.36	0.690762	newly 5 weeks spec	607.	1419.8	{417., 1904.}	149.428	0.590257	newly 5 weeks cumul	1297.	3722.64	{716., 5984.}	204.598	0.662971	newly 66 weeks cumul	9186.	8162.84	{6969., 9339.}	11.8823	0.302555
32	newly 3 weeks spec	258.	179.103	{27., 377.}	52.9404	0.408208	newly 3 weeks cumul	565.	407.259	{63., 915.}	55.5722	0.41217	newly 5 weeks spec	577.	296.707	{60., 555.}	49.2888	0.420242	newly 5 weeks cumul	1557.	942.207	{170., 1951.}	50.1628	0.412101	newly 58 weeks cumul	8222.	7683.6	{7019., 8247.}	6.76843	0.031291
33	newly 3 weeks spec	126.	24.2679	{0., 60.}	81.9586	1.25276	newly 3 weeks cumul	184.	43.625	{1., 121.}	77.6883	1.03783	newly 5 weeks spec	733.	85.1071	{0., 209.}	88.3892	1.62935	newly 5 weeks cumul	1177.	175.911	{1., 392.}	85.3577	1.3626	newly 66 weeks cumul	9187.	6589.64	{1., 9160.}	28.5738	0.783265
34	newly 3 weeks spec	135.	1089.36	{0., 2744.}	786.933	1.89594	newly 3 weeks cumul	190.	4198.88	{0., 8241.}	2189.94	2.24191	newly 5 weeks spec	568.	120.1	{0., 325.}	83.9296	1.91827	newly 5 weeks cumul	1068.	4722.11	{0., 8924.}	422.145	2.12681	newly 68 weeks cumul	8375.	4772.28	{0., 9120.}	46.7279	2.00301
35	newly 3 weeks spec	297.	754.533	{298., 1108.}	162.054	0.447646	newly 3 weeks cumul	580.	1683.45	{568., 2776.}	198.911	0.492525	newly 5 weeks spec	563.	952.683	{631., 1163.}	74.3902	0.293238	newly 5 weeks cumul	1559.	3535.02	{1818., 4856.}	133.012	0.417167	newly 63 weeks cumul	8157.	7744.52	{4777., 9091.}	13.7013	0.139866
36	newly 3 weeks spec	233.	210.661	{32., 452.}	56.0501	0.345046	newly 3 weeks cumul	424.	443.806	{71., 881.}	65.2998	0.350306	newly 5 weeks spec	562.	386.581	{76., 707.}	41.2524	0.301763	newly 5 weeks cumul	1395.	1125.35	{195., 2198.}	48.3524	0.321357	newly 61 weeks cumul	9215.	7934.24	{7154., 8784.}	13.8986	0.0676467
37	newly 3 weeks spec	197.	33.8209	{1., 80.}	82.832	1.13717	newly 3 weeks cumul	298.	68.3433	{4., 175.}	78.2681	0.939646	newly 5 weeks spec	605.	82.8358	{6., 217.}	86.3081	1.19137	newly 5 weeks cumul	1277.	203.881	{13., 516.}	84.0344	1.09734	newly 64 weeks cumul	9224.	7844.87	{6874., 8899.}	14.9619	0.178881
38	newly 3 weeks spec	40.	13.1096	{0., 41.}	84.7603	1.34201	newly 3 weeks cumul	62.	28.2055	{1., 68.}	87.6049	1.00807	newly 5 weeks spec	491.	47.7945	{0., 130.}	92.0291	2.08086	newly 5 weeks cumul	699.	100.507	{1., 279.}	88.6511	1.63708	newly 67 weeks cumul	8849.	5202.67	{1., 9178.}	42.5851	1.34163
39	newly 3 weeks spec	103.	14.8571	{0., 40.}	92.0388	1.73032	newly 3 weeks cumul	146.	26.4857	{1., 59.}	91.4873	1.39332	newly 5 weeks spec	645.	46.3286	{0., 129.}	93.1982	2.25578	newly 5 weeks cumul	1060.	100.171	{1., 288.}	92.7601	1.87408	newly 67 weeks cumul	9368.	5044.26	{1., 8976.}	46.159	1.3857
40	newly 3 weeks spec	276.	690.255	{151., 1245.}	172.2	0.543819	newly 3 weeks cumul	519.	1320.44	{256., 2630.}	177.912	0.529402	newly 5 weeks spec	547.	1094.	{512., 1456.}	112.97	0.449481	newly 5 weeks cumul	1464.	3368.73	{1201., 5103.}	145.594	0.494799	newly 64 weeks cumul	8627.	7778.27	{5379., 9164.}	14.2715	0.203352
41	newly 3 weeks spec	249.	248.155	{24., 546.}	61.5219	0.387152	newly 3 weeks cumul	416.	499.	{49., 1116.}	75.0083	0.387511	newly 5 weeks spec	680.	507.397	{77., 876.}	40.3068	0.344354	newly 5 weeks cumul	1527.	1377.9	{166., 2640.}	49.7889	0.35203	newly 63 weeks cumul	9091.	8200.33	{6873., 9195.}	10.1758	0.0540407
42	newly 3 weeks spec	386.	244.91	{47., 466.}	46.9763	0.371705	newly 3 weeks cumul	794.	541.239	{118., 1008.}	48.5075	0.369734	newly 5 weeks spec	673.	409.239	{111., 655.}	40.4382	0.327741	newly 5 weeks cumul	2037.	1270.94	{331., 2221.}	44.6208	0.355291	newly 58 weeks cumul	8720.	8143.48	{7430., 8543.}	6.6115	0.0303731
43	newly 3 weeks spec	32.	13.44	{0., 24.}	104.563	1.65909	newly 3 weeks cumul	43.	21.26	{0., 40.}	102.465	1.41289	newly 5 weeks spec	315.	86.86	{0., 172.}	99.8286	2.31179	newly 5 weeks cumul	461.	148.02	{0., 260.}	101.228	2.07806	newly 68 weeks cumul	9060.	3815.73	{0., 8989.}	58.2964	2.37722
44	newly 3 weeks spec	255.	170.8	{34., 359.}	52.4947	0.403381	newly 3 weeks cumul	527.	368.108	{67., 808.}	54.9934	0.410482	newly 5 weeks spec	597.	307.477	{107., 610.}	49.9704	0.402243	newly 5 weeks cumul	1543.	908.631	{240., 1879.}	50.5768	0.400659	newly 59 weeks cumul	8693.	8234.17	{7782., 8587.}	5.27816	0.0239747
45	newly 3 weeks spec	293.	370.587	{51., 817.}	77.3606	0.367865	newly 3 weeks cumul	562.	694.095	{88., 1599.}	79.1165	0.374749	newly 5 weeks spec	606.	751.048	{182., 1191.}	52.36	0.241994	newly 5 weeks cumul	1576.	2013.49	{375., 3683.}	65.5064	0.293993	newly 63 weeks cumul	8765.	8401.63	{7033., 9248.}	7.74255	0.036144
46	newly 3 weeks spec	273.	693.919	{373., 880.}	162.123	0.415194	newly 3 weeks cumul	518.	1843.18	{804., 2577.}	263.529	0.539252	newly 5 weeks spec	572.	713.242	{472., 893.}	35.0242	0.137471	newly 5 weeks cumul	1495.	3276.05	{1989., 4075.}	126.476	0.355144	newly 61 weeks cumul	8858.	7158.81	{5516., 8355.}	19.2496	0.0980298
47	newly 3 weeks spec	140.	75.4717	{7., 159.}	60.7547	0.620194	newly 3 weeks cumul	227.	152.528	{14., 333.}	60.7181	0.51546	newly 5 weeks spec	505.	179.472	{29., 401.}	66.2769	0.726982	newly 5 weeks cumul	1027.	449.83	{75., 948.}	61.9279	0.63544	newly 63 weeks cumul	9302.	7795.75	{6618., 8889.}	16.1927	0.203702
48	newly 3 weeks spec	115.	183.667	{0., 445.}	118.719	0.683532	newly 3 weeks cumul	204.	311.474	{1., 811.}	116.581	0.678608	newly 5 weeks spec	483.	598.	{0., 1324.}	76.6372	0.667539	newly 5 weeks cumul	959.	1270.18	{5., 3042.}	86.2064	0.680267	newly 66 weeks cumul	9126.	7098.79	{18., 9342.}	23.0456	0.552634
49	newly 3 weeks spec	85.	13.1096	{0., 41.}	86.9944	1.63131	newly 3 weeks cumul	133.	28.2055	{1., 68.}	87.0327	1.29191	newly 5 weeks spec	380.	47.7945	{0., 130.}	90.8976	1.97422	newly 5 weeks cumul	694.	100.507	{1., 279.}	88.6286	1.63421	newly 67 weeks cumul	8859.	5202.67	{1., 9178.}	42.6066	1.34193
50	newly 3 weeks spec	88.	64.6538	{2., 152.}	66.3899	0.669336	newly 3 weeks cumul	156.	120.154	{7., 291.}	67.6282	0.588217	newly 5 weeks spec	376.	164.788	{3., 375.}	66.1876	0.872177	newly 5 weeks cumul	733.	389.192	{12., 970.}	62.3518	0.699561	newly 65 weeks cumul	9078.	7067.96	{502., 9086.}	22.4173	0.417542
